# Circulating levels and the bioactivity of miR-30b increase during pubertal progression in boys

**DOI:** 10.3389/fendo.2023.1120115

**Published:** 2023-01-19

**Authors:** Nina Mørup, Rytis Stakaitis, Ailsa Maria Main, Ieva Golubickaite, Casper P. Hagen, Anders Juul, Kristian Almstrup

**Affiliations:** ^1^ The Department of Growth and Reproduction, Copenhagen University Hospital - Rigshospitalet, Copenhagen, Denmark; ^2^ The International Center for Research and Research Training in Endocrine Disruption of Male Reproduction and Child Health, Rigshospitalet, Copenhagen, Denmark; ^3^ The Laboratory of Molecular Neurooncology, Neuroscience Institute, Lithuanian University of Health Sciences, Kaunas, Lithuania; ^4^ The Department of Cellular and Molecular Medicine, Faculty of Health and Medical Sciences, University of Copenhagen, Copenhagen, Denmark; ^5^ The Department of Genetics and Molecular Medicine, Lithuanian University of Health Sciences, Kaunas, Lithuania; ^6^ The Department of Clinical Medicine, Faculty of Health and Medical Sciences, University of Copenhagen, Copenhagen, Denmark

**Keywords:** puberty, miRNA, miR-30b, MKRN3, biomarker, HPG-axis

## Abstract

**Background:**

Puberty marks the transition from childhood to adulthood and is initiated by activation of a pulsatile GnRH secretion from the hypothalamus. MKRN3 functions as a pre-pubertal break on the GnRH pulse generator and hypothalamic expression and circulating levels of MKRN3 decrease peri-pubertally. In rodents, microRNA miR-30b seems to directly target hypothalamic *MKRN3* expression – and in boys, circulating levels of miR-30b-5p increase when puberty is pharmacologically induced. Similarly, miR-200b-3p and miR-155-5p have been suggested to inhibit expression of other proteins potentially involved in the regulation of GnRH secretion. Here we measure circulating levels of these three miRNAs as boys progress through puberty.

**Materials and Methods:**

Forty-six boys from the longitudinal part of the Copenhagen Puberty Study were included. All boys underwent successive clinical examinations including estimation of testis size by palpation. miR-30b-5p, miR-200b-3p, and miR-155-5p were measured in serum by RT-qPCR using a kit sensitive to the phosphorylation status of the miRNAs. Thirty-nine boys had miRNA levels measured in three consecutive samples (pre-, peri-, and post-pubertally) and seven boys had miR-30b-5p levels measured in ten consecutive samples during the pubertal transition.

**Results:**

When circulating levels of miR-30b-5p in pre- and peri-pubertal samples were compared with post-pubertal levels, we observed a significant increase of 2.3 and 2.2-fold (p-value<6.0×10^-4^), respectively, and a larger fraction of miR-30b-5p appeared to be phosphorylated post-pubertally indicating an increase in its bioactivity. We also observed a negative correlation between circulating levels of miR-30b-5p and MKRN3. The inter-individual variation in circulating miR-30b levels was substantial and we could not define a clinical threshold for miR-30b-5p suggestive of imminent puberty. Also, miR-155-5p showed significantly increasing levels from the peri- to the post-pubertal stage (p=3.0×10^-3^), whereas miR-200b-3p did not consistently increase.

**Conclusion:**

Both circulating levels of miR-30b-5p and its bioactivity increase during the pubertal transition in boys supporting its role in the activation of the HPG axis at the onset of physiologically normal puberty.

## 1 Introduction

Puberty is the period of transition from childhood to adulthood and is characterized by gonadal maturation, development of secondary sexual characteristics, and gain of reproductive function (1). The reproductive hormones testosterone, follicle-stimulating hormone (FSH), and luteinizing hormone (LH) regulate sexual development, sexual function, and reproduction in men. The circulating levels of reproductive hormones are regulated by feedback loops such as the hypothalamic-pituitary-gonadal (HPG) axis and the initiation of puberty entails (re)activation of the HPG-axis (1). Gonadotropin-releasing hormone (GnRH) is secreted by the KNDy-neurons (kisspeptin/neurokinin B/dynorphin positive neurons) in the arcuate nucleus of the hypothalamus to stimulate the pituitary gland to produce LH and FSH, which in turn stimulate testicular Leydig and Sertoli cells to produce testosterone and inhibin B, and acquire the capacity to support spermatogenesis (2). The HPG-axis is already activated during mini-puberty at 3-6 months of age (1, 3) but is repressed in mid-childhood until puberty is initiated. GnRH inhibitors such as makorin RING-finger protein 3 (MKRN3), Zinc finger E-box-binding homeobox 1 (Zeb1), and CCAAT/enhancer-binding protein beta (Cebpb) play key roles in the repression of GnRH release (2, 4). The complex mechanism behind pubertal onset is still not completely understood (1, 5) and multiple sets of genes, proteins, and microRNAs (miRNAs) seem to be involved in this process (6).

In rodents, MKRN3 inhibits the HPG-axis and pubertal initiation and in humans, loss-of-function mutations in *MKRN3* are linked to central precocious puberty (7–12). In accordance, circulating levels of MKRN3 decrease in boys and girls during the pubertal transition (7, 13). However, the interindividual level of MKRN3 varied substantially (range<25-1285 pg/mL), which excludes it as a clinically useful marker of pubertal transition. Collectively, these studies indicate that MKRN3 functions as a break in the hypothalamic GnRH secretion and that its regulation is crucial for the initiation of puberty.

In rodents, the microRNA miR-30b has been shown to regulate Mkrn3 expression (6). Heras et al. showed that Mkrn3 and miR-30b display opposite expression profiles in the hypothalamus of rats during puberty. Furthermore, if the repressive action of miR-30b on Mkrn3 was interrupted, the decline in *Mkrn3* was not observed and pubertal onset was delayed (6). In accordance with the results from rodents, circulating miR-30b levels have been reported to increase in boys after pharmacological induction of puberty with testosterone or the aromatase inhibitor letrozole in a cohort of 26 boys with delayed onset of puberty (14). These results indicate that circulating levels of miR-30b may represent a novel endocrine signal and regulator of male puberty, albeit confirmatory studies are needed.

Like Mkrn3, Zeb1 and Cebpb are also inhibitors of GnRH secretion and a recent study showed that miRNA-200/429 and miRNA-155 are key components of a developmental switch in miRNA expression which controls the activity of the GnRH promoter in mice. Thus, similarly to miR-30b, increasing levels of miR-200 and miR-155 block the repressive function of Zeb1 and Cebpb, leading to a sustained increase in GnRH release and the initiation of puberty (4). Very little is known about the involvement of miR-200 and miR-155 in the regulation of the human HPG-axis but increasing evidence indicates a profound involvement of miRNAs in the hypothalamic regulation of GnRH secretion and pubertal onset.

For miRNAs to be active, they are phosphorylated at the 5’ end. The presence of non-5’-phosphorylated miRNAs has been suggested to act as a buffer of bioavailability for a fast response to external stimuli (15). This implies that the level of miRNAs in a sample does not necessarily reflect the number of bioactive miRNAs unless the method for miRNA quantification is sensitive to the 5’ phosphorylation. Therefore, the detailed methodological principles of the cDNA synthesis are important and the commonly used kits from Applied Biosystems differ greatly on this account. The TaqMan® Advanced miRNA cDNA synthesis kit states that the 5’ phosphorylation of the miRNAs is required for the RT reaction since an adaptor is ligated to the 5’ end. In contrast, the stem-loop primer of the TaqMan® MicroRNA Reverse Transcription Kit is attached to the 3’ end of the miRNAs and therefore has no preference for whether a 5’ phosphorylation is present or not.

In this study, we measured the circulating levels of miR-30b, miR-200b, and miR-155 in a cohort of 46 healthy boys and identify significant changes during their pubertal transition. We chose only to investigate boys because Varimo et al. (14), who reported changes in miR-30b levels, also focused on boys that were medically stimulated to enter puberty and also to minimize the potential extra variability that may originate from post-pubertal girls starting their hormonal and menstrual cycles.

## 2 Materials and methods

### 2.1 Study population

The Copenhagen Puberty Study (ClinicalTrials.gov ID: NCT01411527) is a combined cross-sectional and longitudinal population-based cohort of healthy Danish children and adolescents conducted from 2006-2014 (16, 17). For the current study, 112 boys participating in the longitudinal part of the study, where they were examined every 6 months, were included, as described in detail by Mouritsen et al. (18). Blood samples were drawn at each examination (between 8 AM and 1 PM) and a clinical examination including testicular volume measurement by orchidometer was conducted (19). Pubertal onset was defined as a uni- or bilateral testicular volume of 4 mL or more. The age at pubertal onset was set as the date exactly between the two visits when the boys passed the mark of pubertal onset. The pubertal age at specific time points was calculated by using the date for blood sampling relative to the calculated date for pubertal onset. Reproductive hormones were measured as described by (20).

**Figure 1:**
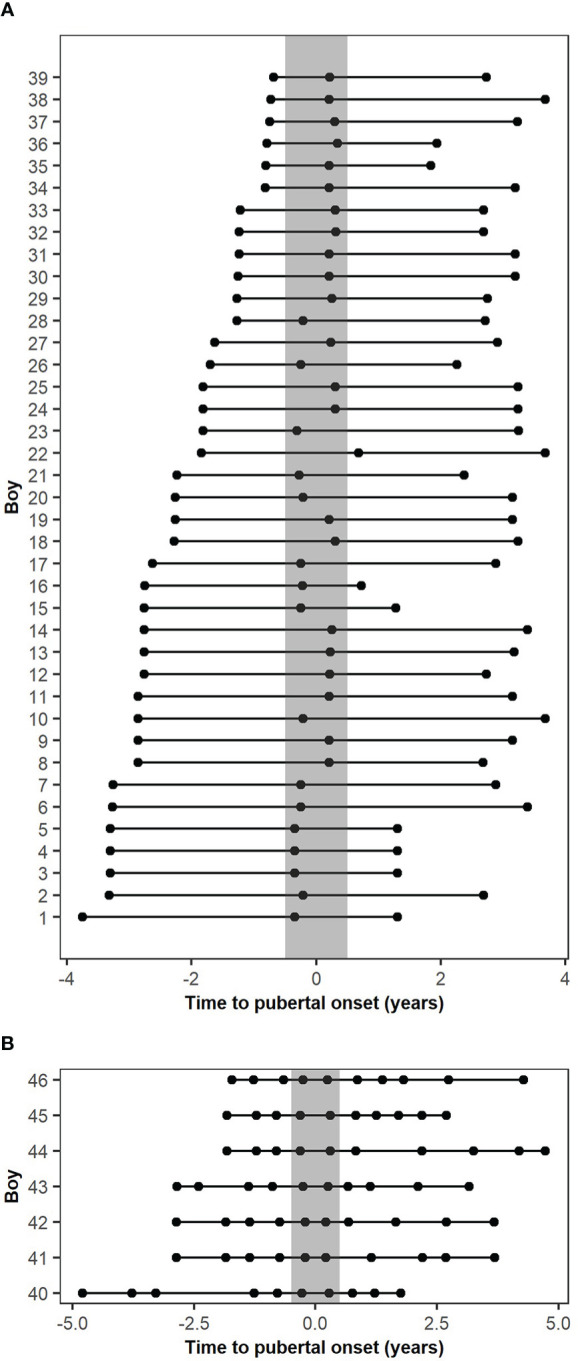
Descriptive figure of the timing of blood sampling according to the pubertal onset of the included cohorts. **(A)** Blood sample timing according to the pubertal onset of the main cohort. **(B)** Blood sample timing according to the pubertal onset of the extended cohort. The grey bar represents pubertal onset +/- 0.5 years.

Boys were excluded from the present study based on the selection criteria described in Busch et al. (7); no blood samples drawn at pubertal onset, one or both parents originating from a non-European country, missing pubertal staging, previous gonadotoxic chemotherapy or cerebral illness, and missing blood sample, leaving 60 boys with measured levels of circulating MKRN3 (7). Of these 60 boys, 12 were excluded due to lack of a blood sample before, during, and/or after puberty, and two were excluded since there were less than two years between the first and last blood samples. Thus, 46 boys were included, and these were divided into two cohorts; the main cohort with 39 boys who had miR-30b levels measured in three consecutive blood samples drawn before (>0.5 years before), during (+/- 0.5 years), and after (>0.5 years after) onset of puberty and an extended cohort with seven boys who had miR-30b levels measured in ten consecutive blood samples drawn during pubertal progression (3-5 samples drawn before (>0.5 years before), 1-2 during (+/- 0.5 years), and 3-5 after (>0.5 years after) pubertal onset) (**Figures 1A, B**). The median age at pubertal onset was 11.57 years (9.88-12.75) for the main cohort and 12.03 years (10.83-13.73) for the extended cohort. Hormone profiles of the main cohort represent normal pubertal progression (**Table 1** and **Supplementary Figure 1**).

**Table 1:** Descriptive table of the main cohort.

Timing of pubertal onset	Pre, N = 39*^1^*	Peri, N = 39*^1^*	Post, N = 39*^1^*
Age at testis transition from 3 mL to 4 mL	11.57 (11.07, 12.01)		
Age (years)	9.40 (8.70, 9.85)	11.70 (11.05, 12.00)	14.40 (13.40, 14.90)
Bilateral testis size (mL)	2.0 (2.0, 2.5)	4.0 (3.0, 4.0)	15.0 (8.0, 20.0)
MKRN3 (pg/mL)	141 (94, 192)	122 (94, 200)	108 (79, 168)
Normalized miR-30b levels (×10^-8^)	0.68 (0.39, 1.41)	0.72 (0.27, 1.33)*	1.58 (0.74, 2.42)
FSH (IU/L)	0.69 (0.39, 1.07)	1.65 (1.28, 2.30)	3.24 (2.23, 4.45)*
LH (IU/L)	0.06 (0.03, 0.10)	0.65 (0.48, 1.24)	2.81 (2.42, 3.42)*
Testosterone (nmol/L)	0 (0, 0)	0 (0, 1)	14 (11, 19)*
AMH (pmol/L)	558 (460, 646)	414 (259, 602)	51 (41, 68)
InhibinB (pg/mL)	94 (71, 108)	172 (142, 212)	230 (188, 268)*
SHBG (nmol/L)	113 (91, 154)	90 (75, 121)	40 (33, 53)*

^1^Median (IQR), * Missing data from one sample. AMH, Anti-Müllerian hormone; FSH, Follicle-stimulating hormone; LH, Luteinizing hormone; MKRN3, Makorin RING-finger protein 3; SHBG, Sex hormone-binding globulin.

### 2.2 Purification of miRNAs

miRNAs were purified from stored serum using the TaqMan^®^ miRNA ABC purification kit – Human Panel A from LifeTechnologies (cat. no. 4473087) following the Manufacturer’s instructions with minor adjustments. For the main cohort (39 boys with three samples), 50 μL serum was lysed with 100 μL ABC buffer combined with 1μL 0.1nM spike-in miRNA (ath-miR159a). 70 μL beads were used per sample for purification, which was then done according to the Manufacturer’s instructions and miRNAs were eluted in 20 μL Elution buffer. For the extended cohort (seven boys with ten samples each), 100 μL serum was lysed with 200 μL ABC buffer combined with 1 μL 0.1 nM spike-in miRNA (ath-miR159a), and 70 μL beads were used per sample. miRNAs were eluted in 20 μL Elution buffer.

### 2.3 cDNA synthesis and RT-qPCR analysis of miRNAs

Two different kits from Applied Biosystems were used for cDNA synthesis and RT-qPCR measurements of miRNA - the Advanced miRNA setup and the stem-loop miRNA setup.

#### 2.3.1 Advanced miRNA setup

Reverse transcription was done using the TaqMan® Advanced miRNA cDNA synthesis kit according to the manufacturer’s instructions with minor adjustments. Briefly, 0.5 μL 10x Poly(A) Buffer, 0.5 μL ATP, 0.3 μL Poly A Enzyme, and 0.5 μL 0.05 nM spike-in miRNA (cel-miR-39) were mixed with 4 μL purified miRNA and placed in a Thermal cycler at 37°C for 45 min and 65°C for 10 min. After this, a mixture of 3μL 5x DNA ligase buffer, 4.5 μL 50% PEG 8000, 0.6 μL 25x Ligation Adaptor, 1.5 μL RNA ligase, and 0.4 μL RNase-free water was added, and the sample was placed in the Thermal cycler at 16°C for 60 min. Then, a cocktail of 6 μL 5x RT buffer, 1.2 μL dNTPs (25mM each), 1.5 μL 20x Universal RT primer, 3 μL RT Enzyme mix, and 3.3 μL RNase-free water was added to the sample and it was incubated in the Thermal cycler at 42°C for 15 min and 85°C for 5 min. The samples were then preamplified by combining 15 μL RT product with 25 μL 2x miR-Amp Master mix, 2.5 μL 20x miR-Amp Primer mix, and 7.5 μL RNase-free water. This was incubated in the Thermal cycler at 95°C for 5 min, 14 cycles of 95°C for 3 sec and 60°C for 30 sec, and then 99°C for 10 min. The RT and pre-amp products were stored at -20°C until used for qPCR. miRNA quantification was performed using the TaqMan® Fast Advanced Master Mix (no UNG) and TaqMan® Advanced miRNA Assays (hsa-miR-30b-5p, hsa-miR-155-5p, hsa-miR-200b-3p, ath-miR159a, and cel-miR-39-3p), according to the manufacturer’s instructions. All samples were run in duplicates in a 96-well PCR plate on a QuantStudio Pro 6.

#### 2.3.2 Stem-loop miRNA setup

Reverse transcription was done using the TaqMan® MicroRNA Reverse Transcription Kit according to the manufacturer’s instructions with minor adjustments using either a primer pool containing a mix of primers specific for miR159a, miR-155, miR-30b, and miR-200b (for analysis of serum samples) or single primers for miR159a or miR-30b (for test for phosphorylation of specific miRNAs). Shortly, an RT primer pool was made with ath-miR159a, ipu-miR-155 (targeting hsa-miR-155-5p), hsa-miR-30b (targeting hsa-miR-30b-5p), and hsa-miR-200b (targeting hsa-miR-200b-3p) in a final 0.05X concentration. 6 μL RT primer pool (0.05X) or a single RT primer (5X) was mixed with 0.3 μL 100mM dNTPs, 3 μL MultiScribe™ Reverse Transcriptase, 1.5 μL 10X Reverse Transcription Buffer, 0.19 μL RNase Inhibitor, 1.01 μL Nuclease-free water, and 3 μL purified miRNAs, placed on ice for 5 min, and run on a thermal cycler under the following settings: 16°C for 30 min, 42°C for 30 min, and 85°C for 5 min. Pre-amplification was run in a thermocycler after mixing 2.5 μL of the RT reaction with 12.5 μL TaqMan® Fast Advanced MasterMix, no UNG, 6.25 μL Nuclease-free water, and 3.75 μL 0.2X pre-amplification primer pool (ath-miR159a, ipu-miR-155, hsa-miR-30b, and hsa-miR-200b) or primer (ath-miR159a or hsa-miR-30b) with the following settings: 95°C for 10 min, 55°C for 2 min, 72°C for 2 min, 12 cycles of 95°C for 15 s and 60°C for 4 min, and 99.9°C for 10 min. miRNA quantification was performed using the TaqMan® Fast Advanced Master Mix (no UNG) and TaqMan® MicroRNA Assays (ath-miR159a, ipu-miR-155, hsa-miR-30b, and hsa-miR-200b) according to manufacturer’s instructions. All samples were run in duplicates in a 96-well PCR plate on a QuantStudio Pro 6.

### 2.4 miRNA data analysis

miR159a was used as a control for purification efficiency and samples with levels deviating from the other samples. miR-39 was analyzed as extra quality control with the Advanced setup when miR159a levels deviated. If the miR159a levels in the individual samples deviated largely from the rest, the sample was reanalyzed. To account for differences in purification efficiency, raw miR-30b Ct values were normalized to the miR159a levels from the same sample by subtracting the difference in miR159a Ct value for the sample compared with the average miR159a Ct value for the whole plate by the following equation Ct_norm_=Ct_hsa-miR30b_-(Ct_ath-miR159a_-average(Ct_ath-miR159a_plate_) and transformed for the further analyses (2^-Ctnorm^).

### 2.5 MKRN3 measurements

MKRN3 levels were measured for a previous study (7) and the method is described by Hagen et al. (13). Shortly, MKRN3 levels were measured using the commercially available human makorin ring finger protein 3 ELISA (MyBioSource). The value of 12.5 pg/mL (0.5 times the detection limit) was assigned as the level of MKRN3 for measurements below the detection limit.

### 2.6 Data analysis

Data analysis was performed using R (21) version 4.1.2 and RStudio version 2021.09.2 + 382. Data were prepared for statistical analysis using magrittr (22), tidyverse (23), dplyr (24), and psych (25), and statistics (Wilcoxon tests with Bonferroni correction) comparing miRNA levels in pre-, peri-, and post-pubertal samples were calculated using rstatix (26).

## 3 Results

### 3.1 Changes in circulating miRNAs during puberty

**Figure 2:**
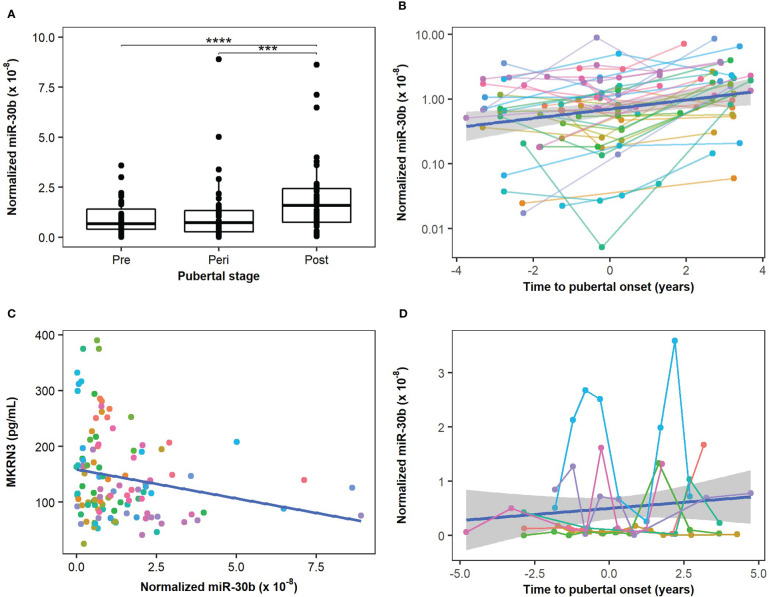
Circulating miR-30b levels during the pubertal transition. Circulating levels of miR-30b were measured in boys from the main cohort (n=39) and compared in groups representing pre-, peri, and post-pubertal states. **(A)** A statistically significant increase in circulating miR-30b levels was found when pre- and peri-pubertal states were compared with post-pubertal states (p=1.21×10^-5^ and 6×10^-4^, respectively). **(B)** The circulating miR-30b levels in the main cohort were plotted according to the estimated pubertal timing of each sample and a trendline indicating an increase in circulating miR-30b levels during the pubertal transition is shown in blue. The colors represent individual boys and matched samples from the same boy are connected with lines matching the color of the dots. **(C)** Correlation between measured levels of miR-30b in the main cohort and circulating protein levels of Makorin RING-finger protein 3 (MKRN3) measured in the same samples (previously described in (7)). Adjusted R^2^: 0.035, correlation coefficient: -0.21, p-value: 0.025. **(D)** Measurements of miR-30b-5p in 10 successive samples during the pubertal transition of seven boys representing the extended cohort were plotted according to the estimated pubertal timing of each sample and a trendline indicating a gradual increase in miR-30b is shown in blue. The colors represent individual boys and matched samples from the same boy are connected with lines matching the color of the dots. The plot reveals a substantial individual variation (average CV 151%) in circulating miR-30b levels during the pubertal transition. ***p < 10^-3^ and ****p < 10^-4^.

We measured the level of miR-30b, miR-155, and miR-200b in serum from 39 healthy boys with longitudinally paired samples representing pre-, peri- and post-pubertal states (**Figure 1A**) using the Advanced miRNA setup. Circulating levels of miR-30b (potentially regulating *MKRN3*) were found at significantly higher levels post-pubertally when compared with both pre- and peri-pubertal states (p=1.2×10^-5^ and p=6.0×10^-4^, respectively; **Figure 2A**). In addition, when each sample was plotted according to the estimated pubertal timing (i.e., not grouped) a trendline indicated gradually increasing levels of miR-30b during the pubertal transition (**Figure 2B**).

Circulating levels of MKRN3 were previously measured in the same samples (7) and when we compared circulating levels of miR-30b with protein levels of MKRN3 we found that miR-30b levels were inversely correlated with MKRN3 levels (**Figure 2C**). Albeit the correlation was not strong (correlation value -0.21, adjusted R^2^ value 0.035), these data indicate a potential suppressive effect of miR-30b on *MKRN3* expression.

We noticed a substantial variation in the individual pubertal trajectories of miR-30b (**Figure 2B**) and therefore performed a more detailed pubertal profiling of seven children with 10 samples each during the pubertal transition. The extended cohort also revealed an increase in miR-30b during pubertal progression and a great intraindividual variation (CV 67%-233%) was evident (**Figure 2D**).

Circulating miR-200b-3p levels (potentially regulating *ZEB1*) did not show significant changes between the pubertal states (**Supplementary Figures 2A, B**), whereas circulating levels of miR-155-5p (potentially regulating *CEBPB*) showed a significant change only from peri- to post-pubertal states (p=0.003) and not between pre- and post-pubertal states (**Supplementary Figures 2C, D**).

### 3.2 The 5’-phosphorylation status of miR-30b changes during the pubertal progression

**Figure 3:**
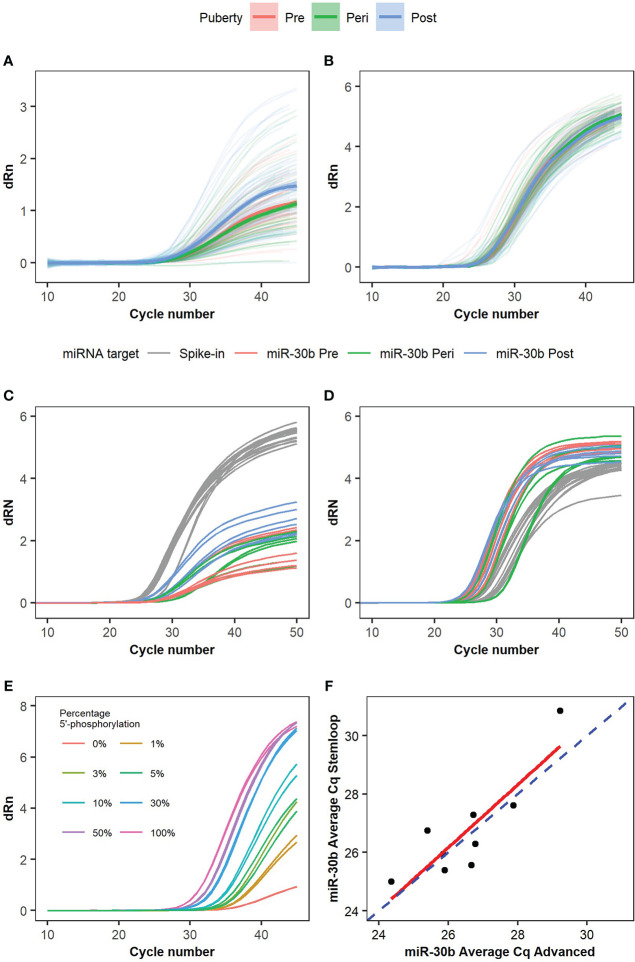
Ambiguous amplification curves and phosphorylation level of miR-30b. Amplification curves for **(A)** miR-30b and **(B)** spike-in control miR159a were analyzed in samples from the main cohort using the Advanced setup. n=39 boys with 3 samples each. Trendlines with confidence intervals indicating the average amplification curve for pre- (red), peri- (green), and post-pubertal (blue) samples are shown, and the individual curves are seen in the background with faded, matched colors according to the pubertal stage. Nine samples were measured with both **(C)** the Advanced miRNA setup and **(D)** the stem-loop miRNA setup and the amplification curves are colored according to target (grey for spike-in independent of the pubertal state, red for miR-30b in pre-pubertal samples, green for miR-30b in peri-pubertal samples, and blue for miR-30b in post-pubertal samples). **(E)** Amplification curves for synthetic miR-30b with and without 5’-phosphorylation measured by RT-qPCR using the Advanced miRNA setup. Different percentages of miR-30b with 5’-phosphorylation were mixed with miR-30b without 5’-phosphorylation to investigate the change in the shape of the curves depending on the percentage of fully functional and 5’-phosphorylated miRNAs in the sample. The maximum dRn value is visibly lower when only 10% of the miRNAs are 5’-phosphorylated but a rising curve is still observed when only non-phosphorylated (inactive) miRNAs are present although no exponential phase is seen. **(F)** Correlation plot comparing the miR-30b Cq values of the nine samples using the advanced miRNA setup vs. the stem-loop miRNA setup. The dashed blue line represents x=y, the red line represents the linear model for the correlation of the two setups. Individual dots represent the nine samples analyzed. Correlation coefficient: 0.86, Adjusted R^2^: 0.7033, p-value: 0.0029.

Inspection of the qPCR amplification curves for miR-30b (**Figure 3A**) using the Advanced miRNA setup revealed striking differences in the maximum dRn value (fluorescence signal from the qPCR run) that the individual curves reached. In contrast, the amplification curves for the spike-in miR159a had the same shapes and reached equivalent maximum dRn values (**Figure 3B**). Since the Advanced miRNA setup requires a 5’-phosphorylation of the miRNAs for efficient cDNA synthesis, we questioned whether the differences in the maximum dRn could be related to differences in the 5’-phosphorylation status of miR-30b.

To further test the impact of the 5’-phosphorylation, we tested a subset of the samples with a kit (the Stem-loop miRNA setup), which works independently of the 5’-phosphorylation. We selected nine samples from three boys from the main cohort and analyzed miR-30b and the spike-in miR159a with both the Advanced and the Stem-loop setups. The amplification curves for miR159a analyzed by both setups looked similar and reached a maximum dRn above 4 after 50 cycles (**Figures 3C, D**, grey curves). In contrast, the amplification curves for miR-30b again showed very different maximum dRn values with the different kits (maximum dRn: 1-3 with the Advanced miRNA setup and >4 with the Stem-loop miRNA setup, **Figures 3C, D**). This indicates that the effect on dRn is specific to the endogenous miR-30b and the Advanced setup.

We further tested this by measuring different fractions of synthetic miR-30b with and without 5’-phosphorylation by qPCR. This revealed a striking dose-response relationship between the fraction of synthetic miR-30b with a 5’-phosphorylation level and the maximum dRn value (**Figure 3E**), where higher fractions of 5’-phosphorylated miR-30b led to higher maximum dRn values.

Using the synthetic spike-in miR159a with and without a 5’-phosphorylation further revealed that the Advanced miRNA kit indeed can measure both phosphorylated and unphosphorylated miRNAs (**Supplementary Figure 3A**). However, the efficiency differs depending on the phosphorylation status of the miRNA. Fully phosphorylated miR159a reaches a high maximum dRn value and the different dilutions have the expected effect on the Cq-values (shifts to the right in an exponential manner). In contrast, the non-phosphorylated miR159a showed a pattern recapitulating miR-30b with a lower maximum dRn value and more flat amplification curves. Again, the Stem-loop setup shows no major differences between the amplification curves (**Supplementary Figure 3B**). Hence, we were able to mimic the observed effects on miR-30b by altering the 5’-phosphorylation on miR159a.

The effect, however, mainly seems to affect the maximum dRn value and has only a slight effect on the Cq-values, as the Cq-values showed a significant (p=0.003) correlation when comparing the two kits (Adjusted R^2^ 0.7; **Figure 3F**).

## 4 Discussion

**Figure 4:**
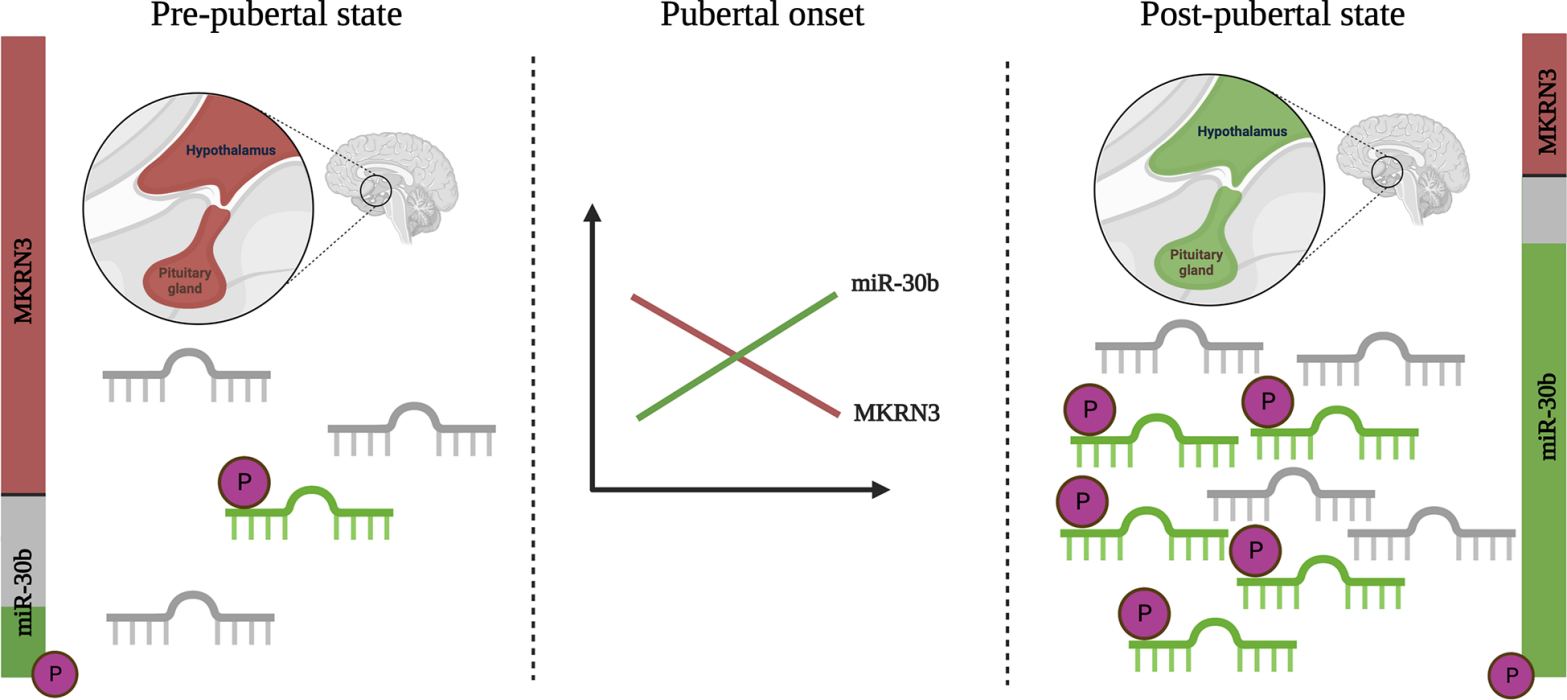
Schematic illustration of changes in miR-30b levels and bioactivity during pubertal transition. Circulating levels of miR-30b are low in pre-pubertal boys and a large fraction of these appears unphosphorylated at the 5’ end (inactive, shown in grey). At the same time, levels of Makorin RING-finger protein 3 (MKRN3) in circulation are high (shown in red) in pre-pubertal boys. As puberty progresses, levels of miR-30b increase and a larger fraction appear to be biologically active with a 5’-phosphorylation (shown in green). A concurrent drop in the circulating level of MKRN3 is observed. While the data builds on measurements in blood, we believe that these, at least to some extent, reflect hypothalamic activation of the kisspeptin/neurokinin B/dynorphin positive (KNDy-) neurons, which are known from studies in animals to be involved in the onset of puberty. Created with BioRender.com.

In this longitudinal study of healthy boys followed through pubertal development, we found a significant increase in circulating levels of miR-30b as boys progress through puberty. A similar increase of circulating miR-30b levels has previously been described by Varimo et al. in boys medically stimulated to enter puberty (14). Both our study and the study by Varimo et al. found great inter- and intra-individual variation in miR-30b levels, but still saw an overall significant increase in miR-30b levels after pubertal onset (>6 months after pubertal onset or start of medication). Studies in rats report that miR-30b represses *Mkrn3* transcription in the hypothalamus (6) and we believe that circulating levels of miR-30b in boys, at least during puberty, can serve as a proxy for hypothalamic miR-30b levels. In support of this, we found an inverse correlation between circulating levels of miR-30b and MKRN3 in boys during the pubertal transition. As illustrated in **Figure 4**, we speculate that peri-pubertal changes of miR-30b cause a down-regulation of MKRN3 and a subsequent release of the brake on the GnRH production. In addition, we identified 5’-phosphorylation of miR-30b as a potential novel physiological regulatory mechanism of the miR-30b bioactivity. Prepubertally, we found both low levels of miR-30b in circulation and a large proportion of it without 5’-phosphorylation, whereas post-pubertally, we found higher circulating miR-30b levels with a larger proportion of it appearing as 5’-phosphorylated, which is needed for the bioactivity of the molecule (**Figure 4**). The increase in miR-30b levels seem to be delayed compared with the decrease observed in circulating MKRN3 levels, which is already evident between 5 to 3 years prior to pubertal onset (7). This could indicate that the inhibition of MKRN3 expression is regulated by additional factors besides miR-30b or that the changes in miR-30b expression levels inside the hypothalamic neurons are not reflected in circulation until later during the pubertal transition.

Although the study from rats on the involvement of miR-30b in the activation of the HPG-axis is quite clear and specific (6), the human miR-30 family is also described to be expressed in multiple tissues (according to the human miRNA tissue atlas) and to be involved in many physiological processes. miR-30b is among the 119 miRNAs most commonly found in human serum and plasma (27) and has been used as a stable reference miRNA both in cohorts of adult men (e.g. men with testicular cancer (28)) and in cohorts of pediatric patients (29). Recently, miR-30b was also described in lower levels in the ejaculate of men with poor semen quality compared with fertile men (30), which indicates a potential link to testicular function. However, due to the multi-tissue expression of miR-30b it is unknown if circulating levels of miR-30b, represent secretion from other organs in addition to the hypothalamic expression. In addition, according to miRDB (mirdb.org), miR-30b targets 19 different transcripts (including *MKRN3*) with a perfect match (target score of 100). Interestingly, one of the other predicted targets of miR-30b is *LIN28B*, which is linked to both age of menarche in girls and age of voice break in boys (31). Hence, besides *MKRN3*, miR-30b also targets a substantial number of other transcripts with high affinity, and consequently, miR-30b most likely serves other functions than regulating hypothalamic *MKRN3* levels. Indeed, miR-30b has been described to both inhibit and promote cell proliferation, migration, invasion, and mesenchymal transformation of cancer cells depending on the type and has well-described roles in cardiovascular and metabolic disease (32). Interestingly, several members of the miR-30 family (miR-30a to e) seem to target *MKRN3*, which in total contains three miR-30 target sites, and this implies that some redundancy among miR-30 family members is expected. It could be interesting to measure the level of all miR-30 members during the pubertal transition and deduce their physiological origin.

During our study, we noticed that the shape of the amplification curves for miR-30b varied a lot from sample to sample. Strikingly, the amplification curves reaching the highest dRn were predominantly from the post-pubertal samples, whereas the curves with the lowest maximum dRn were predominantly from the pre- and peri-pubertal samples. We found that the changes in the amplification curves mimicked the differences observed when running the analysis with different proportions of phosphorylated and unphosphorylated miRNA spike-ins. This indicated that there could be a difference in the amount of 5’-phosphorylation, which is normally needed for a miRNA to be biologically active (15). Surprisingly, the use of the Advanced miRNA setup added another layer to the study by taking the 5’-phosphorylation status into account. To further investigate this, we also used the Stem-loop miRNA setup, which functions independently of the 5’-phosphorylation. With the Stem-loop miRNA setup, there was no difference in the shape of the curves according to pubertal status but the Ct values correlated well with the Advanced miRNA setup. Collectively, we believe that the data on 5’-phosphorylation of miR-30b intensify the effect of the peri-pubertal increase by adding an increase in its bioactivity. It has previously been shown that irradiation of cancer cell lines caused a pool of miR-34 without a 5’-phosphorylation to become phosphorylated, relocate from the nucleus to the cytoplasm, and be loaded into the Argonaute complex. The phosphorylation of miR-34 was dependent on the serine-protein kinase ATM and the RNA kinase Clp1 and likely serve as an acute response to irradiation (15). However, to our knowledge, our study is the first to show that the 5’-phosphorylation of a miRNA has a potential regulatory mechanism of a normal physiological change like puberty.

Besides miR-30b, we also investigated miR-200b and miR-155 but only identified a statistical difference between peri- and post-pubertal levels of miR-155. Whether this is because the hypothalamic expression of miR-200b and miR-155 and their regulatory effect on Zeb1 and Cebpb is less profound than miR-30b or whether we are simply underpowered to detect differences remains to be explored. Interestingly, miR-200b levels have previously been described to be changed in several diseases including endometriosis (33, 34), ovarian cancer (35), as well as in prostate (36) and cervical (37) carcinomas, which are all at least partially hormone-dependent conditions. This could suggest some linkage of miR-200b expression to changing hormone levels as observed during puberty. In addition to the three miRNAs measured in this study, other factors also influence pubertal progression like genetic variants in specific genes like *MKRN3* and *DLK1*, which have been linked to precocious puberty in both boys and girls (8, 38). Future studies focusing on other small RNAs could include small RNA sequencing of serum from boys and girls throughout pubertal transition, instead of the targeted approach that we have used. This could also open for new functional studies to get a better understanding of the molecular mechanisms regulating puberty.

Albeit, the Copenhagen Puberty cohort represents a clinically very well-described puberty cohort, providing a unique set of samples throughout the pubertal transition, the sample size remains rather small. Furthermore, the samples were collected from 2006 to 2010 and have thus been stored for 12-16 years before being used for this study. We also observed a substantial intra-individual variation in the miR-30b levels – similar to what have previously been reported for MKRN3 levels in the same cohort (7). Since the GnRH pulse generator entails a pulsative action, such variation could be physiologically important but could also represent technical and sample variation.

Future studies should try to validate our findings of increasing miR-30b levels during puberty and deduce the functional consequences of the 5’-phosphorylation of miR-30b. Including a larger cohort of boys and more samples during their pubertal transition could help in working out whether any increase in circulating miR-30b or a switch in the 5’-phosphorylation can be detected prior to what we observed, e.g., between 5 and 3 years before pubertal onset (7). Finally, albeit the hormonal cyclic nature of post-pubertal girls likely makes it difficult to measure miR-30b post-pubertally, in future studies it would be very interesting to see whether the circulating miR-30b levels increase peri-pubertally in girls and whether there are any differences in the miR-30b levels and their 5’-phosphorylation status of girls going through normal, precocious and delayed puberty, especially because a recent study has indicated that hypothalamic overexpression of Mkrn3 causes delayed puberty in female, but not male mice (39).

In conclusion, we show that circulating levels of miR-30b-5p as well as its potential bioactivity increase during the pubertal transition in boys. Albeit a substantial intra-individual variation was observed, our results support a role for miR-30b in the activation of the HPG axis initiating pubertal onset.

## Data availability statement

The datasets analyzed for this study can be requested from the authors at a reasonable request. Requests to access these datasets should be directed to Kristian Almstrup, kristian.almstrup@regionh.dk.

## Ethics statement

The studies involving human participants were reviewed and approved by the local ethical committee of the Capital Region of Denmark. Written informed consent to participate in this study was provided by the participants’ legal guardian/next of kin.

## Author contributions

NM performed the experimental work, collected, analyzed, and interpreted the data, carried out the statistical analyses, and drafted, reviewed, and revised the manuscript. RS performed experimental work, designed the study, analyzed and interpreted the data, and reviewed and revised the manuscript. AM drafted, reviewed, and revised the manuscript. IG performed experimental work, and reviewed and revised the manuscript. CH collected and interpreted the data, and reviewed and revised the manuscript. AJ supervised the study, interpreted the data, and reviewed and revised the manuscript. KA conceptualized, designed, and supervised the study, interpreted data, and drafted, reviewed, and revised the manuscript. All authors approved the final manuscript.
