# Supplementary material for: Circulating levels and the bioactivity of miR-30b increase during pubertal progression in boys
Source: Front Endocrinol (Lausanne). 2023 Jan 19;14:1120115. doi: 10.3389/fendo.2023.1120115 (PMC9893272; doi:10.3389/fendo.2023.1120115)
Supplement: Supplementary file 1 [file DataSheet_1.docx]

Supplementary Material

# Supplementary Data

## Supplementary Figures


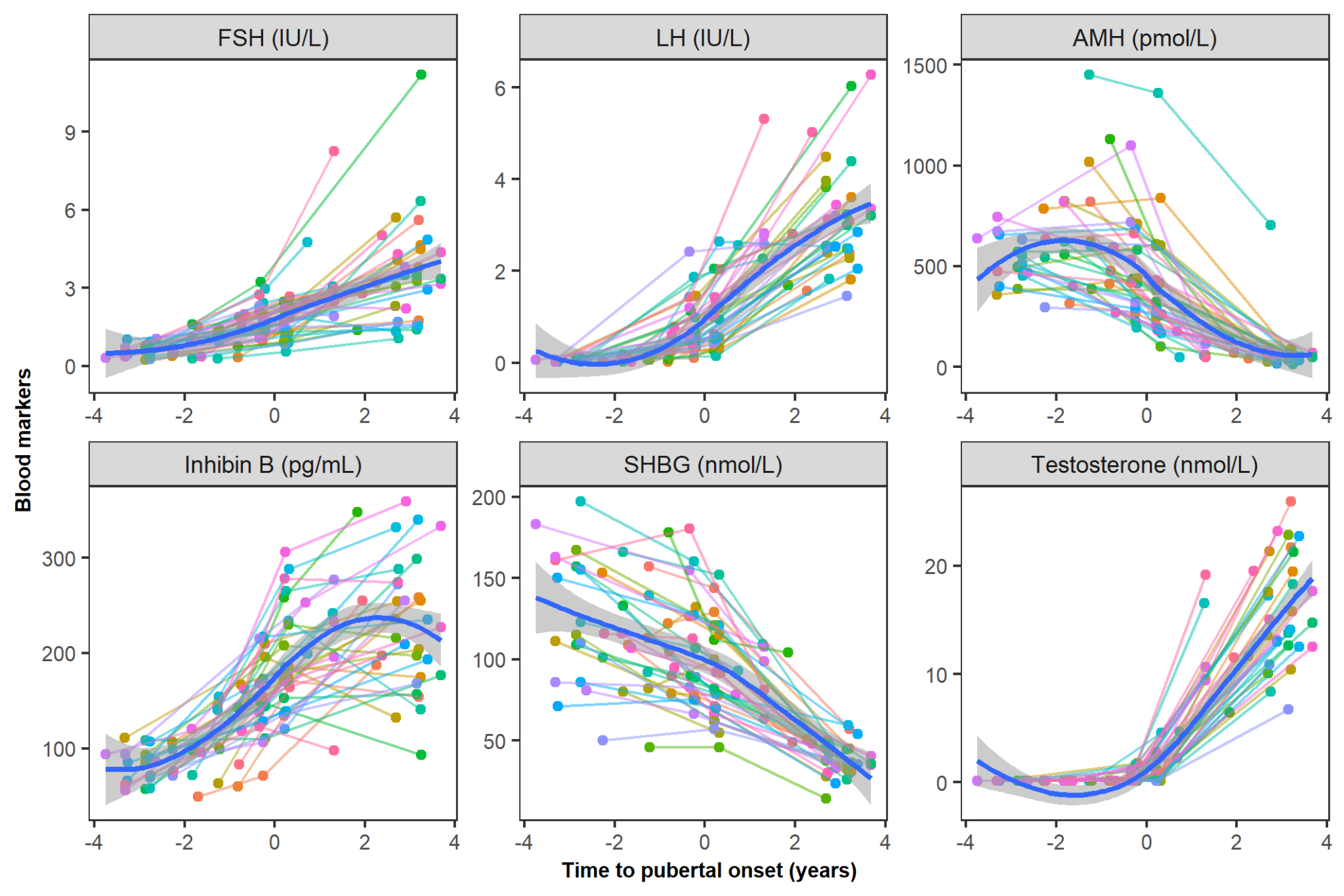


**Supplementary Figure 1.** Hormone profiles of the boys from the main cohort. Hormone profiles of the boys during pubertal transition support the pubertal staging of the boys and the estimated time of pubertal onset. Parts of the data have previously been published as parts of Busch et al 2016, Mouritzen et al 2013, Mouritzen et al 2014, Sørensen et al 2010, Aksglæde et al 2010, Greiber et ak 2018, Lindhardt Johansen et al 2014 and Mieritz et al 2015 (1–8). AMH, Anti-Müllerian hormone; FSH, Follicle-stimulating hormone; LH, Luteinizing hormone; SHBG, Sex hormone-binding globulin.


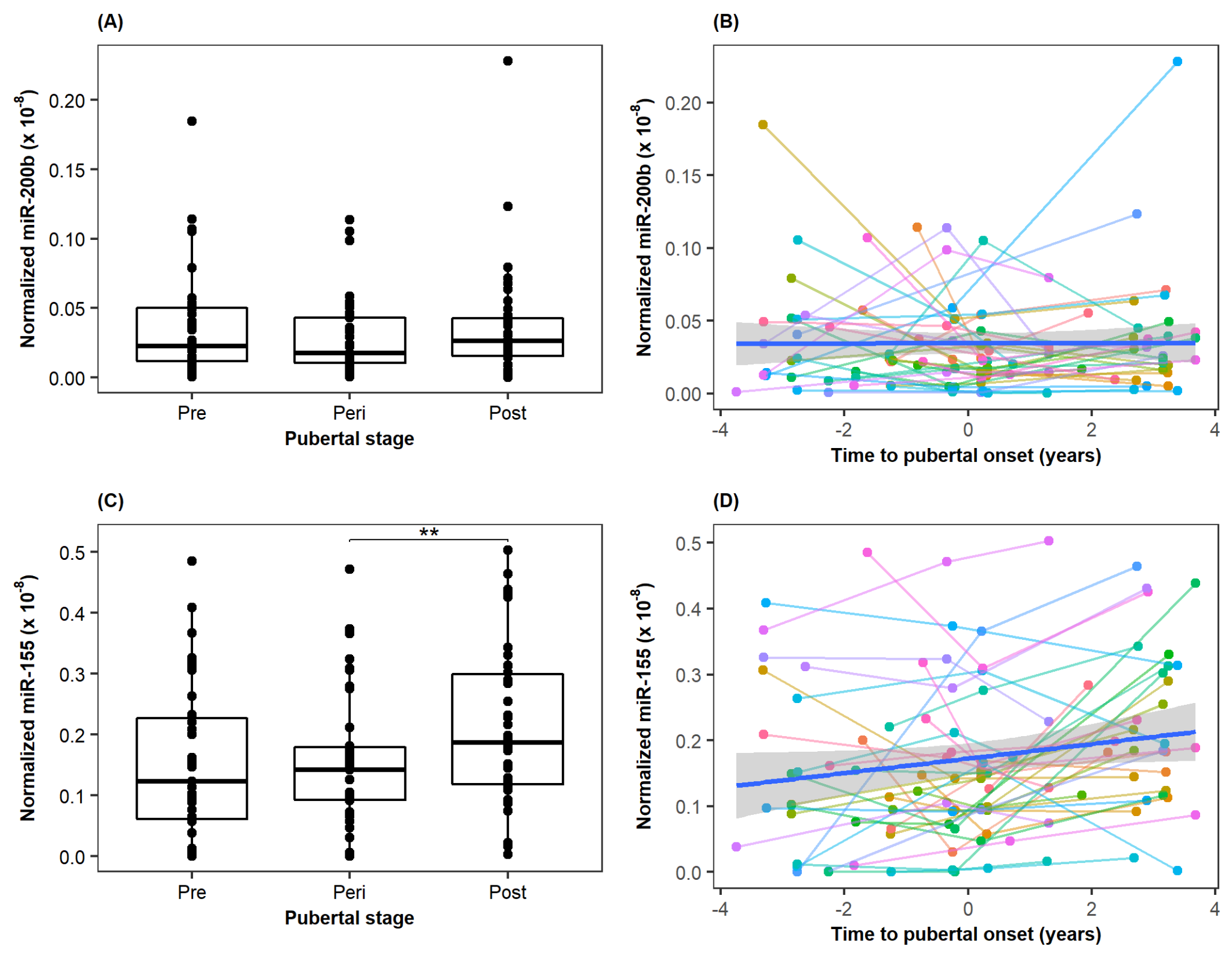


**Supplementary Figure 2.** Circulating miR-200b and miR-155 levels in boys from the main cohort. **(A)** Circulating miR-200b levels in the main cohort according to the pubertal stage reveals no significant differences. **(B)** Individual circulating miR-200b levels for each boy with connecting lines for matched samples and a trendline for miR-200b plotted according to the estimated time to pubertal onset showing no difference in circulating levels according to pubertal timing. **(C)** Circulating miR-155 levels in the main cohort according to the pubertal stage reveals a significant difference between peri- and post-pubertal samples (p-value: 0.003). **(D)** Individual circulating miR-155 levels for each boy with connecting lines for matched samples and a trendline for miR-155 plotted according to the estimated time to pubertal onset showing an increase in circulating levels during pubertal progression.

## Supplementary Figure 3


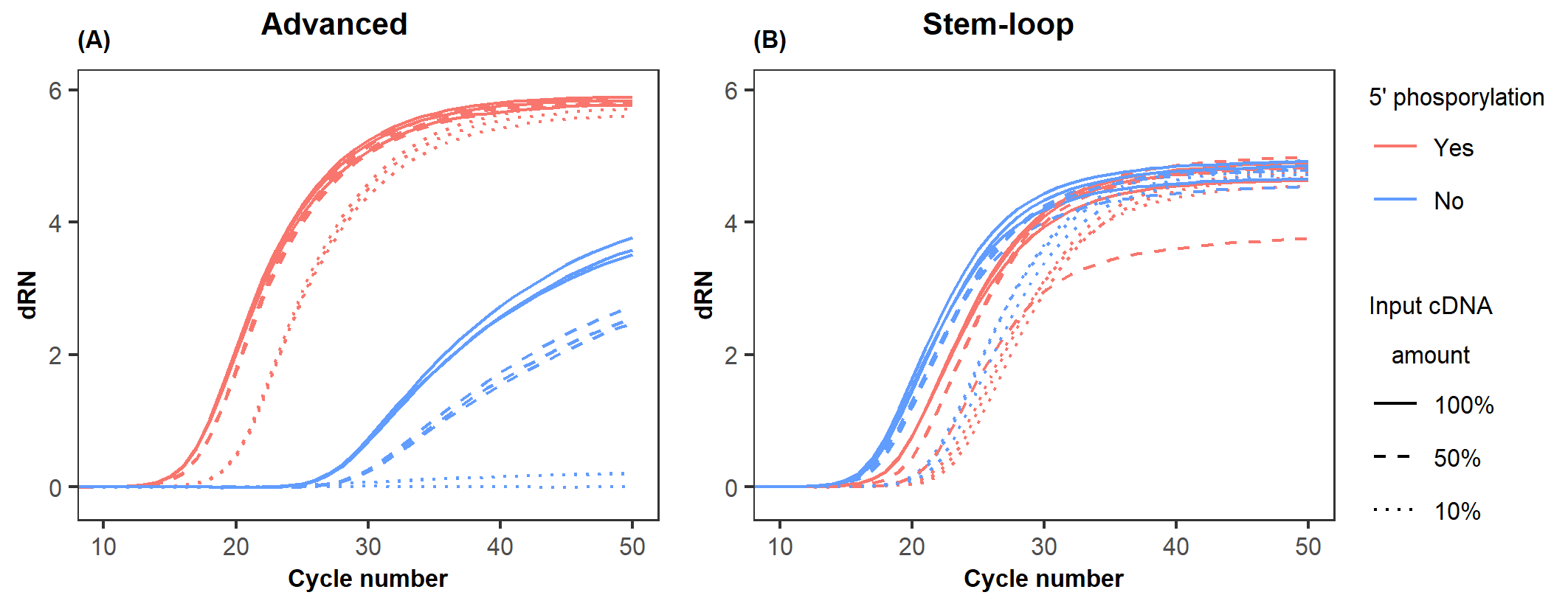


**Supplementary Figure 3. qPCR analysis of the spike-in miRNA miR159a with and without 5’-phosphorylation.** Amplification curves using **(A)** the Advanced miRNA setup and **(B)** the stem-loop miRNA setup for miR159a with (red) and without (blue) 5’-phosphorylation measured at three different concentrations (100% shown as a line, 50% shown as a dashed line and 10% shown as a dotted line). Dilution series for the 5’-phosphorylated miRNAs with both setups show the expected shift to the right representing exponential growth whereas non-phosphorylated miR-30b only shows the expected shift with the stem-loop miRNA setup, whereas the curves are generally shifted to the right at all concentrations with the advanced miRNA setup and both the differences according to the dilutions (not representing exponential growth) and the shape of the curves (representing the efficiency of the PCR run) differ greatly from the results with the phosphorylated miRNAs.

# References for Supplementary Figures

1. Busch AS, Hagen CP, Almstrup K, Juul A. Circulating MKRN3 levels decline during puberty in healthy boys. *J Clin Endocrinol Metab* (2016) 101:2588–2593. doi: 10.1210/jc.2016-1488

2. Mouritsen A, Søeborg T, Johannsen TH, Aksglaede L, Sørensen K, Hagen CP, Mieritz MG, Frederiksen H, Andersson AM, Juul A. Longitudinal changes in circulating testosterone levels determined by LC-MS/MS and by a commercially available radioimmunoassay in healthy girls and boys during the pubertal transition. *Horm Res Paediatr* (2014) 82:12–17. doi: 10.1159/000358560

3. Mouritsen A, Aksglaede L, Soerensen K, Hagen CP, Petersen JH, Main KM, Juul A. The pubertal transition in 179 healthy Danish children: associations between pubarche, adrenarche, gonadarche, and body composition. *Eur J Endocrinol* (2013) 168:129–36. doi: 10.1530/EJE-12-0191

4. Sørensen K, Aksglaede L, Petersen JH, Juul A. Recent changes in pubertal timing in healthy Danish boys: Associations with body mass index. *J Clin Endocrinol Metab* (2010) 95:263–270. doi: 10.1210/jc.2009-1478

5. Aksglaede L, Sørensen K, Boas M, Mouritsen A, Hagen CP, Jensen RB, Petersen JH, Linneberg A, Andersson AM, Main KM, et al. Changes in Anti-Müllerian Hormone (AMH) throughout the life span: A population-based study of 1027 healthy males from birth (cord blood) to the age of 69 years. *J Clin Endocrinol Metab* (2010) 95:5357–5364. doi: 10.1210/jc.2010-1207

6. Greiber IK, Hagen CP, Busch AS, Mieritz MG, Aksglæde L, Main K, Almstrup K, Juul A. The AMH genotype (rs10407022 T>G) is associated with circulating AMH levels in boys, but not in girls. *Endocr Connect* (2018) 7:347–354. doi: 10.1530/EC-17-0299

7. Mieritz MG, Rakêt LL, Hagen CP, Nielsen JE, Talman MLM, Petersen JH, Sommer SH, Main KM, Jørgensen N, Juul A. A longitudinal study of growth, sex steroids, and IGF-1 in boys with physiological gynecomastia. *J Clin Endocrinol Metab* (2015) 100:3752–3759. doi: 10.1210/jc.2015-2836

8. Johansen ML, Anand-Ivell R, Mouritsen A, Hagen CP, Mieritz MG, Søeborg T, Johannsen TH, Main KM, Andersson AM, Ivell R, et al. Serum levels of insulin-like factor 3, anti-Müllerian hormone, inhibin B, and testosterone during pubertal transition in healthy boys: A longitudinal pilot study. *Reproduction* (2014) 147:529–535. doi: 10.1530/REP-13-0435
